# Ferumoxytol potentiates ferroptotic radiosensitization through NCOA4-associated iron mobilization in brain-metastatic lung cancer cells

**DOI:** 10.3389/fphar.2026.1883092

**Published:** 2026-10-14

**Authors:** Ziqiang Shen, Jiangrui Zhu, Yongliu Yang, Jing Yang, Lisha Rao, Chong Zhou, Youyou Xia

**Affiliations:** 1 Department of Radiation Oncology, The Affiliated Lianyungang Hospital of Xuzhou Medical University/The First People’s Hospital of Lianyungang, Lianyungang, Jiangsu, China; 2 Department of Radiation Oncology, The First People’s Hospital of Lianyungang/Lianyungang Clinical College of Nanjing Medical University, Lianyungang, Jiangsu, China; 3 Lianyungang Medical-Education Innovation and Research Center, Nanjing Medical University, Lianyungang, Jiangsu, China; 4 Department of Radiation Oncology, Southeast University Affiliated Xuzhou Central Hospital, Xuzhou, Jiangsu, China

**Keywords:** brain metastasis, ferroptosis, ferumoxytol, iron metabolism, lung cancer, NCOA4, radiosensitization

## Abstract

**Background:**

Brain metastasis remains a major cause of mortality in patients with advanced non-small cell lung cancer, whereas effective radiosensitization strategies are constrained by heterogeneous tumor responses and the tolerance of normal brain tissue. Ferumoxytol, a clinically used iron oxide nanoparticle formulation, has emerging translational potential as an iron-modulating agent. However, whether Ferumoxytol enhances radiosensitivity in brain-metastatic lung cancer cells and the mechanisms underlying this effect remain unclear.

**Methods:**

PC-9 lung adenocarcinoma cells and NCI-H1915 brain-metastatic lung adenocarcinoma cells were used to evaluate ferumoxytol under basal and irradiated conditions. Ferroptosis-associated phenotypes, oxidative stress, clonogenic survival, and cell-cycle distribution were assessed. TMT-based proteomics and public single-cell and spatial transcriptomic analyses were used for candidate prioritization and disease-context support. NCOA4 was evaluated by siRNA-mediated loss of function, single-dose clonogenic assays, time-resolved NCOA4/FTH1 immunoblotting, bafilomycin A1-based autophagic-flux analysis, and endogenous NCOA4-FTH1 co-immunoprecipitation.

**Results:**

Ferumoxytol induced mitochondrial, biochemical, and molecular changes consistent with ferroptosis and enhanced irradiation-associated ROS accumulation, lipid peroxidation, and loss of clonogenic survival. Proteomic analysis prioritized NCOA4, while public transcriptomic analyses provided associative disease-context support. In H1915 cells, NCOA4 silencing attenuated combined-treatment Fe^2+^ accumulation, oxidative stress, and γ-H2AX induction. The incremental reduction in colony formation produced by adding ferumoxytol to irradiation was also reduced after NCOA4 knockdown in H1915 cells and was no longer significant in PC-9 cells. Time-resolved immunoblotting showed marked FTH1 accumulation rather than net FTH1 depletion, with a late reduction in NCOA4. Bafilomycin A1 caused further LC3B-II and SQSTM1/p62 accumulation under IR + F, consistent with ongoing autophagic turnover, whereas co-immunoprecipitation showed greater normalized recovery of FTH1 in NCOA4 immunoprecipitates after IR + F.

**Conclusion:**

Ferumoxytol enhances ferroptosis-associated oxidative injury and radiosensitivity in the tested lung cancer cell models. The combined loss-of-function, autophagic-flux, and protein-interaction data support the involvement of NCOA4-associated ferritin handling and iron mobilization. However, the observed FTH1 accumulation and the absence of iron-chelator rescue, cargo-specific lysosomal tracking, and *in vivo* validation preclude a definitive claim of canonical ferritinophagy or clinical efficacy.

## 1 Introduction

Lung cancer, especially NSCLC, is one of the most common and lethal malignancies worldwide (Leiter et al., 2023). Brain metastases are frequent and clinically devastating complications of advanced NSCLC and are associated with poor outcomes. Radiotherapy remains a major local treatment for brain metastases from lung cancer (Schiff et al., 2022; Weller et al., 2024). However, in clinical practice, a substantial proportion of brain metastatic lesions show either primary or acquired radioresistance, which limits local control and intracranial progression-free survival (Bou Dargham et al., 2025). Because of the dose tolerance of normal brain tissue, simply escalating the radiation dose is unlikely to further improve efficacy and may markedly increase the risk of radiation necrosis and cognitive impairment (Yarlagadda et al., 2026). Therefore, it is of considerable clinical importance to develop biological radiosensitization strategies that enhance tumor response without increasing injury to normal brain tissue.

Ferroptosis is an iron-dependent form of regulated cell death driven by phospholipid peroxidation (Dixon and Olzmann, 2024). It is mechanistically distinct from apoptosis and necrosis. Hallmark features include oxidative damage to polyunsaturated fatty-acid phospholipids, impairment of the glutathione/GPX4 antioxidant axis, and expansion of the labile iron pool (Guo et al., 2025; Patel and Galvan, 2025; Mao et al., 2026). Ferroptosis is increasingly implicated in tumor progression, treatment resistance, and immune escape; conversely, increasing ferroptotic susceptibility may enhance anticancer treatment (Wahida and Conrad, 2025; Zhang et al., 2026a). Ionizing radiation (IR) produces direct DNA double-strand breaks and ROS through water radiolysis (Lei et al., 2021; Zhang et al., 2026b). It can also promote lipid peroxidation, impair redox homeostasis, and suppress the system Xc^−^/GSH/GPX4 axis through pathways that include ATM-SLC7A11 signaling (Koppula et al., 2017; Liu et al., 2022; He et al., 2023; Fantone et al., 2024). Tumor cells may counter these effects through NRF2-dependent antioxidant programs, lipid remodeling, and altered iron transport (Koppula et al., 2022; Shi et al., 2024; Li et al., 2025). Such adaptation may contribute to radioresistance.

Among the factors that regulate ferroptosis, intracellular iron homeostasis and its dynamic control appear to be particularly important (Chen et al., 2020; Galy et al., 2023). Labile Fe^2+^ catalyzes the conversion of H_2_O_2_ into highly reactive hydroxyl radicals through Fenton chemistry, thereby promoting oxidative injury and lipid peroxidation (Dixon and Olzmann, 2024). The reversible transition of iron from storage forms, such as ferritin, to the LIP is highly dependent on the autophagy-lysosome system. Ferritinophagy, a selective form of autophagy, promotes lysosomal degradation of ferritin and releases iron ions, thereby increasing the LIP and amplifying ferroptotic signaling (Hayashima et al., 2021). Nuclear receptor coactivator 4 (NCOA4), a key receptor for ferritinophagy, is thought to play an important role in intracellular iron redistribution and ferroptosis regulation (Wu et al., 2023; Le et al., 2024). Although the relationship between ferritinophagy and treatment sensitivity has attracted increasing attention, it remains unclear whether iron homeostasis remodeling contributes to the regulation of radiotherapy response in the specific setting of brain metastasis from lung cancer, and whether NCOA4 serves as a critical mediator in this process. A recent precision-oncology review further emphasizes that NCOA4-mediated ferritinophagy lies at the intersection of ferritin turnover, labile-iron regulation, ferroptosis, and therapeutic resistance (Chen et al., 2026).

Ferumoxytol is an intravenous iron formulation composed of a superparamagnetic iron oxide nanocrystal core (Fe_3_O_4_/Fe_2_O_3_) coated with a carboxymethyl dextran shell (Long et al., 2024). Ferumoxytol is approved by the US Food and Drug Administration for the treatment of iron deficiency anemia in adults who have intolerance to oral iron or an unsatisfactory response to oral iron, or who have chronic kidney disease. In recent years, with the development of nanomedicine and drug repurposing research, the potential value of Ferumoxytol in cancer diagnosis and therapy has received increasing attention. On the one hand, it can be used as a magnetic resonance imaging (MRI)-related nanoprobe for tumor tracking (Strauss et al., 2026). On the other hand, as an exogenous iron source, Ferumoxytol can be taken up by cells and gradually release iron ions in acidic environments, thereby affecting intracellular oxidative stress and iron metabolism (Chang et al., 2025; Wang et al., 2025). Previous studies have shown that in some malignant tumor models, Ferumoxytol markedly increases intracellular Fe^2+^ levels and, when combined with ROS-generating strategies such as radiotherapy or pharmacological ascorbate, induces substantial DNA single- and double-strand breaks and tumor cell death (Petronek et al., 2023; Singhania et al., 2025). In contrast, in normal stem cell systems such as hematopoietic stem cells, Ferumoxytol may exhibit catalase-like ROS-scavenging activity, reduce H_2_O_2_ burden under stress conditions such as chemotherapy or irradiation, and promote hematopoietic recovery (Wang et al., 2025). These findings suggest that Ferumoxytol may be a promising candidate with both translational potential and radiosensitizing capacity.

However, direct evidence for the role of Ferumoxytol in radiotherapy for brain metastasis from lung cancer is still lacking. In particular, under radiation-induced oxidative stress, it remains unclear whether the exogenous iron load provided by Ferumoxytol can further disrupt tumor iron homeostasis, amplify lipid peroxidation, and promote ferroptosis. It is also unknown whether key molecular nodes determine the radiosensitizing effect during this process. Because dose escalation for brain metastases is constrained by the tolerance of normal brain tissue, and because ferroptosis is highly sensitive to iron homeostasis and lipid peroxidation, systematic investigation of Ferumoxytol-mediated iron homeostasis remodeling and its interaction with radiotherapy may provide important insight into the development of safe and clinically translatable radiosensitization strategies.

Based on these considerations, the present study integrated cell-based functional assays, TMT-based proteomic profiling, public single-cell and spatial transcriptomic analyses, and NCOA4 loss-of-function validation to investigate whether Ferumoxytol enhances irradiation-induced ferroptosis in lung cancer brain metastasis-related models. We hypothesized that Ferumoxytol may amplify radiation-induced oxidative injury by remodeling intracellular iron homeostasis and that NCOA4-associated iron mobilization may serve as a key mechanistic link between exogenous iron loading and ferroptotic radiosensitization. This study aimed to provide a mechanistic basis for further evaluation of Ferumoxytol as a clinically translatable radiosensitizing strategy in lung cancer brain metastasis.

## 2 Materials and methods

This *in vitro* mechanistic study integrated cell-based functional assays, TMT-based proteomic screening, public single-nucleus and spatial transcriptomic analyses, and targeted NCOA4 loss-of-function experiments. PC-9 and NCI-H1915 cells were used to evaluate ferumoxytol-associated ferroptosis and radiation response. Experimental analyses included NCOA4 knockdown in both cell lines, single-dose clonogenic assays after NCOA4 knockdown, time-resolved NCOA4 and FTH1 immunoblotting, BafA1-based assessment of autophagic turnover, endogenous NCOA4–FTH1 co-immunoprecipitation, and cell-cycle analysis. Public transcriptomic data were used as associative disease-context support and not as a substitute for experimental perturbation.

### 2.1 Cell lines and culture

The human lung adenocarcinoma cell line PC-9 and the human brain metastatic lung adenocarcinoma cell line NCI-H1915 were purchased from Shanghai Zhong Qiao Xin Zhou Biotechnology Co., Ltd. (ZQXZBio, Shanghai, China; PC-9: Cat. No. ZQ0487; NCI-H1915: Cat. No. ZQ1144). After receipt, both cell lines were authenticated by short tandem repeat (STR) profiling and tested for *mycoplasma* contamination. PC-9 cells were cultured in DMEM supplemented with 10% fetal bovine serum (FBS; Gibco) and 1% penicillin-streptomycin. NCI-H1915 cells were cultured in RPMI-1640 supplemented with 10% FBS and 1% penicillin-streptomycin. All cells were maintained in a humidified incubator at 37 °C with 5% CO_2_. Cells in the logarithmic growth phase were used for all experiments.

### 2.2 Main reagents and antibodies

Ferumoxytol was purchased from MedChemExpress (MCE, United States of America), dissolved in sterile phosphate-buffered saline (PBS) to prepare a stock solution, and stored at 4 °C protected from light. Ferrostatin-1 (Fer-1), a ferroptosis inhibitor, was also purchased from MCE, dissolved in dimethyl sulfoxide (DMSO), and stored at −20 °C. The final concentration of DMSO in the experiments was kept at ≤0.1%. The Cell Counting Kit-8 (CCK-8) was obtained from Beyotime (China). The DCFH-DA probe for ROS detection was purchased from MCE. Lipid peroxidation was assessed using the C11-BODIPY 581/591 probe kit (Beyotime). Intracellular Fe^2+^ was detected using the FerroOrange probe (Dojindo, Japan). Details of antibodies and dilution ratios are listed in Supplementary Table S1. Bafilomycin A1 (BafA1) was used to inhibit late-stage autophagic degradation, and Protein A/G magnetic beads were used for co-immunoprecipitation. Antibodies against NCOA4, FTH1, LC3B, SQSTM1/p62, γ-H2AX, and GAPDH, together with their working dilutions, are listed in Supplementary Table S1.

### 2.3 Treatment conditions and grouping

To determine the appropriate concentration of Ferumoxytol, cells were first treated with different concentrations of Ferumoxytol for 24 h or 48 h, and cell viability was measured by CCK-8 assay. Based on the dose-response results, a concentration with biological activity but without excessive nonspecific cytotoxicity was selected for subsequent experiments. For ferroptosis inhibition experiments, Fer-1 was added 1 h before the corresponding treatment at a final concentration of 5 μM. The basic groups included the control group (Ctrl), Ferumoxytol group (F), irradiation group (IR), irradiation plus Ferumoxytol group (IR + F), and irradiation plus Ferumoxytol plus Fer-1 group (IR + F + Fer-1). For autophagic-flux analysis, H1915 cells were assigned to Ctrl, IR, IR + F, and IR + F + BafA1 groups; BafA1 was used under the following verified condition: Bafilomycin A1 (BafA1, 0.6 nM) was added during the final 4 h before sample collection, and cells were collected 24 h after irradiation. For time-course analysis, H1915 cells were collected at 0, 6, 12, 24, and 48 h after IR + F treatment.

### 2.4 Irradiation conditions

Irradiation was delivered using a Varian medical linear accelerator with 6 MV X-rays. Cell culture plates were placed on water-equivalent material and covered with a 0.5-cm silicone bolus to ensure dose uniformity. The irradiation parameters were as follows: 6 MV X-rays, source-to-skin distance (SSD) of 100 cm, field size of 20 cm × 20 cm, and dose rate of approximately 400 MU/min. For clonogenic assays, cells were irradiated with 0, 1, 2, 4, and 6 Gy to generate dose-survival curves. For most mechanistic experiments, 4 Gy was used as the working dose. For DNA damage assays, cells were exposed to 8 Gy and collected 2 h later for γ-H2AX detection to evaluate early radiation-induced DNA double-strand breaks. The 8 Gy condition was used only for early DNA damage assessment and was not included in clonogenic survival curve fitting.

### 2.5 Cell viability assay

Cells were seeded into 96-well plates at 5 × 10^3^ cells/well in 100 μL of complete medium and allowed to attach overnight. After the indicated treatments, 10 μL of CCK-8 solution was added directly to each well. The plates were incubated at 37 °C for 1 h, and absorbance was measured at 450 nm (A_450_) using a microplate reader. Wells containing the corresponding culture medium and CCK-8 solution without cells were used as blank controls. When necessary, drug-containing blank wells without cells were included to assess potential interference from the treatment compounds. Cell viability was calculated as follows: [(A_450_ of treated wells − A_450_ of blank wells)/(A_450_ of control wells − A_450_ of blank wells)] × 100%. Each group included at least three technical replicates, and all experiments were independently repeated at least three times.

### 2.6 Transmission electron microscopy (TEM)

After the indicated treatments, cells were collected and fixed in 2.5% glutaraldehyde at 4 °C overnight, followed by postfixation in 1% osmium tetroxide. Samples were dehydrated through a graded ethanol series and embedded in Epon resin. Ultrathin sections were stained with uranyl acetate and lead citrate and examined by transmission electron microscopy. Images were acquired at nominal magnifications of ×5,000 and 10,000×. Scale bars were generated from the original microscope calibration metadata. Images were cropped without altering their aspect ratios, and no nonlinear resizing was applied. Ultrastructural features associated with ferroptosis included reduced mitochondrial volume, decreased or absent cristae, and increased membrane density (Chen et al., 2021).

### 2.7 Detection of ROS, lipid peroxidation, and Fe^2+^

ROS: At 6 h after irradiation, or at the indicated time points after drug treatment, cells were incubated with DCFH-DA (final concentration, 10 μM) at 37 °C for 30 min according to the experimental design. After washing with PBS, the mean fluorescence intensity (MFI) was detected by fluorescence microscopy using the FITC channel.

Lipid peroxidation: Lipid peroxidation was measured using the C11-BODIPY 581/591 probe (2 μM, 37 °C for 30 min), and the ratio of oxidized (green) to reduced (red) fluorescence was analyzed.

Fe^2+^: Intracellular labile Fe^2+^ was detected using the FerroOrange probe (1 μM, 37 °C for 30 min), and fluorescence intensity was compared among groups.

### 2.8 Clonogenic assay and radiosensitization analysis

After irradiation, cells were seeded into 6-well plates at dose-dependent densities. For PC-9 cells, the numbers were 200 cells/well at 0 Gy, 200 at 1 Gy, 400 at 2 Gy, 800 at 4 Gy, and 1,200 at 6 Gy. For H1915 cells, the numbers were 2000 cells/well at 0 Gy, 2000 at 1 Gy, 4,000 at 2 Gy, 8,000 at 4 Gy, and 12,000 at 6 Gy. After the indicated irradiation doses, cells were cultured for 14 days, fixed with 4% paraformaldehyde, and stained with crystal violet. Colonies containing at least 50 cells were counted as surviving colonies (Brix et al., 2021). The surviving fraction (SF) was calculated accordingly. Dose-survival curves were fitted using the single-hit multi-target model (Franken et al., 2006):

$$SF=1-{\left(1-e^{-D/D_{0}}\right)}^{N}$$

where D0 represents the mean lethal dose and N represents the extrapolation number. The quasi-threshold dose (Dq) was calculated as:

$$Dq=D0\times \ln\left(N\right)$$

The sensitizer enhancement ratio (SER) was calculated using the IR group as the reference according to Dq values:

$$SER=\frac{Dq\left(IR\right)}{Dq\left(IR+treatment\right)}$$

Three replicate wells were set for each dose point, and all experiments were independently repeated at least three times.

For NCOA4 loss-of-function experiments, a separate single-dose assay was performed at 4 Gy. After transfection with negative-control siRNA (NC) or siNCOA4, PC-9 and H1915 cells were seeded at 100 and 1,000 cells per well, respectively, and assigned to IR or IR + F treatment. Colonies containing at least 50 cells were counted after 14 days. Because only a single radiation dose was tested, these data are reported as colony formation rate (%) rather than fitted sensitizer enhancement ratios. Three independent experiments were performed.

### 2.9 Western blot

After treatment, cells were washed twice with pre-chilled PBS and lysed in RIPA buffer supplemented with protease and phosphatase inhibitors on ice for 30 min. Lysates were centrifuged at 12,000 × g for 15 min at 4 °C, and the supernatants were collected. Protein concentration was measured using the BCA assay. Equal amounts of total protein (20 μg per lane) were mixed with loading buffer, denatured at 95 °C for 10 min, separated by 10%–12% SDS-PAGE, and transferred onto PVDF membranes. Membranes were blocked with 5% nonfat milk in TBST for 1 h at room temperature; for γ-H2AX detection, 5% BSA in TBST was used instead. Membranes were then incubated overnight at 4 °C with the indicated primary antibodies. The next day, membranes were washed three times with TBST for 10 min each and incubated with HRP-conjugated secondary antibodies for 1 h at room temperature. After washing, protein bands were visualized using an enhanced chemiluminescence substrate and captured using a gel imaging system. Band intensities were quantified using ImageJ software. PC-9 and H1915 samples were analyzed on separate blots. For each independent experiment, target-protein signals were normalized to the corresponding GAPDH signals from the matched samples on the respective blot. Representative immunoblots are shown, and quantitative analyses were based on at least three independent experiments.

For the NCOA4/FTH1 time course, H1915 cells were analyzed at 0, 6, 12, 24, and 48 h after IR + F treatment. FTH1 and NCOA4 band intensities were normalized to GAPDH and expressed relative to the untreated control. For autophagic-flux analysis, LC3B-II and SQSTM1/p62 were quantified relative to GAPDH. The additional accumulation of LC3B-II and SQSTM1/p62 in the presence of BafA1 was interpreted as evidence of ongoing autophagic turnover, but not as cargo-specific proof of ferritin degradation.

### 2.10 Co-immunoprecipitation

H1915 cells from the Ctrl and IR + F groups were collected 24 h after irradiation. Equal amounts of clarified whole-cell lysate were incubated with an anti-NCOA4 antibody or a species-matched IgG control and captured using Protein A/G magnetic beads. After washing, the bound proteins were eluted and analyzed by immunoblotting for FTH1 and NCOA4, with an input fraction analyzed in parallel. For quantitative comparison, co-immunoprecipitated FTH1 was normalized to immunoprecipitated NCOA4, and the control value was set to 1. Each experiment was independently repeated three times.

### 2.11 siRNA transfection and knockdown validation

siRNAs targeting NCOA4 and negative control siRNA were synthesized by GenePharma (Shanghai, China). Among the three candidate siRNAs tested, NCOA4-Homo-1656 showed the highest knockdown efficiency and was used for subsequent experiments. Cells were transfected at 50%–60% confluence using GenePharma RNAi-Mate reagent according to the manufacturer’s instructions. Protein and RNA samples were collected at the indicated time points for knockdown validation. The siRNA sequences are provided in Supplementary Table S2. The validated siRNA was applied to both H1915 and PC-9 cells. H1915 was used for the principal Fe^2+^, ROS, lipid-peroxidation, DNA-damage, and clonogenic analyses, while PC-9 was used as a non-brain-metastatic NSCLC comparison model for knockdown validation, γ-H2AX analysis, and single-dose clonogenic assessment.

### 2.12 qRT-PCR

Total RNA was extracted using a commercial total RNA extraction reagent (NCM Biotech, China), and RNA concentration and purity were assessed by spectrophotometry. Complementary DNA was synthesized using a reverse-transcription kit (Takara Bio Inc., Japan) according to the manufacturer’s instructions. The reverse-transcription reaction was performed in a final volume of 10 μL containing 1 μL of total RNA, 2 μL of reverse-transcription reagent, and 7 μL of nuclease-free water. Quantitative real-time PCR was performed using a SYBR Green qPCR master mix (Takara Bio Inc., Japan) in a final reaction volume of 10 μL containing 5.0 μL of SYBR premix, 3.6 μL of nuclease-free water, 1.0 μL of cDNA, and 0.2 μL of each primer. The amplification protocol consisted of an initial denaturation at 95 °C for 5 min, followed by 40 cycles of denaturation at 95 °C for 10 s and annealing/extension at 60 °C for 30 s. Melting-curve analysis was performed to confirm amplification specificity. Relative mRNA expression was calculated using the 2^−^ΔΔCt method, with GAPDH as the reference gene (Livak and Schmittgen, 2001). Primer sequences are provided in Supplementary Table S3. All experiments were independently repeated at least three times.

### 2.13 TMT proteomics and bioinformatics analysis

H1915 cells were selected for proteomic analysis. After treatment with irradiation alone (IR, 4 Gy) or irradiation combined with ferumoxytol (IR + F, 4 Gy plus 250 μM ferumoxytol), protein samples were collected 48 h after irradiation. Two biological replicates were included for each group. Samples were labeled using tandem mass tags and subjected to LC–MS/MS analysis by Shanghai Applied Protein Technology Co., Ltd. The fold change (FC) was calculated as the protein abundance in the IR + F group divided by that in the IR group. Differentially expressed proteins were defined as those with a fold change (FC) > 1.5 or <0.667 and P < 0.05. KEGG pathway enrichment analysis was then performed. Differentially expressed proteins were further intersected with the ferroptosis-related molecule set in the FerrDb V2 database to identify candidate regulatory factors (Zhou et al., 2023). Protein-protein interaction (PPI) network analysis was subsequently used to identify key nodes (Szklarczyk et al., 2023). Details of differentially expressed proteins are provided in Supplementary Table S4.

### 2.14 Flow cytometry (cell cycle analysis)

For cell cycle analysis, cells were collected at the indicated time points after irradiation or other treatments according to the experimental design and fixed overnight in 70% pre-chilled ethanol. After fixation, cells were washed with PBS and stained with PI/RNase A staining solution (KeyGEN BioTECH, China) for 30 min in the dark. DNA content was analyzed using a BD FACSCanto II flow cytometer (BD Biosciences, United States of America), and the proportions of cells in the G0/G1, S, and G2/M phases were fitted using ModFit LT 5.0 software. At least 1 × 10^5^ events were acquired for each sample. All experiments were independently repeated at least three times.

### 2.15 Public datasets and single-cell transcriptomic analysis

Public transcriptomic datasets related to lung cancer brain metastasis were obtained from the GEO SuperSeries GSE223503, including the single-nucleus RNA-seq dataset GSE223499 and the matched Slide-seq2 spatial transcriptomic dataset GSE223501. For single-nucleus transcriptomic analysis, raw feature-barcode matrices from all available samples were loaded and matched to the corresponding metadata. Cells were filtered according to the original metadata and subjected to quality control, including removal of low-quality cells with low gene counts and cells with high mitochondrial transcript proportions. The filtered data were normalized to a total count of 1 × 10^4^ per cell, log-transformed, and processed for highly variable gene selection. Principal component analysis was then performed in a memory-efficient chunked manner, followed by neighborhood graph construction and UMAP visualization. Cell-type annotation was based on the original metadata labels, including tumor, tumor (not tumor), myeloid, microglia, endothelial, fibroblast, CNS-related, and lymphoid populations. Cells were grouped according to sample origin, including primary tumors and brain metastases. Expression patterns of HMOX1, IREB2, and NCOA4 were visualized by UMAP feature plots, dot plots, and violin plots in both the full atlas and tumor-cell subsets. Differential comparisons between primary and brain metastatic groups were performed using nonparametric tests as described below.

### 2.16 Spatial transcriptomic analysis and cell-type mapping

For spatial transcriptomic analysis, Slide-seq2 coordinate and count matrices from GSE223501 were processed on a per-sample basis and then merged into a combined spatial dataset. Spatial beads were subjected to quality control, including filtering by minimum detected genes, minimum total counts, and mitochondrial transcript proportion. After normalization and log transformation, highly variable genes were identified and used for dimensionality reduction, neighborhood graph construction, UMAP visualization, and Leiden clustering. To infer cellular composition in spatial data, reference cell-type profiles were generated from the single-nucleus RNA-seq dataset using shared genes between the single-cell and spatial datasets. Spatial cell types were then assigned on the basis of similarity scoring to these reference profiles, and the highest-scoring cell type was recorded as the predicted cell type for each spatial spot/bead. Representative samples were further used for spatial visualization of predicted cell types, neighborhood enrichment analysis, and lesion-level expression mapping of HMOX1, IREB2, and NCOA4. In addition, Moran’s I was used to assess spatial autocorrelation of variable genes in representative samples.

### 2.17 In silico perturbation-like analysis, differential expression, and enrichment analyses

To further explore the functional relevance of ferroptosis-associated genes in the metastatic context, an *in silico* perturbation-like, expression-stratified strategy was applied to the brain metastasis tumor-cell subset. Brain metastasis cells were first restricted to tumor-related populations, including Tumor and Tumor (Not Tumor) cells. For each target gene (HMOX1, IREB2, and NCOA4), cells were stratified according to expression status. When sufficient non-expressing cells were present, cells were grouped into gene-positive and gene-negative subsets; otherwise, a median-based expression split was used. Differential expression analysis between the high-expression and low-expression groups was performed using the Wilcoxon rank-sum method implemented in Scanpy. Differentially expressed genes were summarized by volcano plots, DEG counts, and ferroptosis-focused heatmaps based on a curated FerrDb-related ferroptosis gene set. Functional enrichment analysis of significantly altered genes was then performed using GO Biological Process and KEGG pathway libraries. These *in silico* analyses were interpreted as hypothesis-supporting computational evidence and were used to complement, rather than replace, the direct loss-of-function experiments performed *in vitro*.

### 2.18 Statistical analysis

All experimental data are presented as mean ± standard deviation (SD) from at least three independent experiments unless otherwise stated. Comparisons between two groups were performed using a two-tailed unpaired Student’s t-test. Comparisons among multiple groups were performed using one-way ANOVA followed by Tukey’s multiple-comparison test. For experiments involving two independent variables, two-way ANOVA followed by Sidak’s multiple-comparison test was used when appropriate. Clonogenic survival curves were fitted by nonlinear regression using the single-hit multi-target model. Public single-cell and spatial transcriptomic analyses were performed using nonparametric tests implemented in the bioinformatic workflow, with multiple-testing adjustment where applicable. A P value <0.05 was considered statistically significant.

## 3 Results

### 3.1 Ferumoxytol induces ferroptosis-associated phenotypes in lung cancer cells and brain metastatic lung cancer cells

To evaluate the biological effects of Ferumoxytol on PC-9 and H1915 cells, relative cell viability was measured by CCK-8 assay after treatment with different concentrations for 24 h and 48 h. Ferumoxytol reduced the viability of both cell lines, and this effect became more pronounced with increasing concentration and longer exposure time (Figure 1A). Based on the dose-response curves obtained from the CCK-8 assay, 250 μM Ferumoxytol, which produced a moderate inhibitory effect, was selected as the standard working concentration for all subsequent *in vitro* experiments.

**FIGURE 1:**
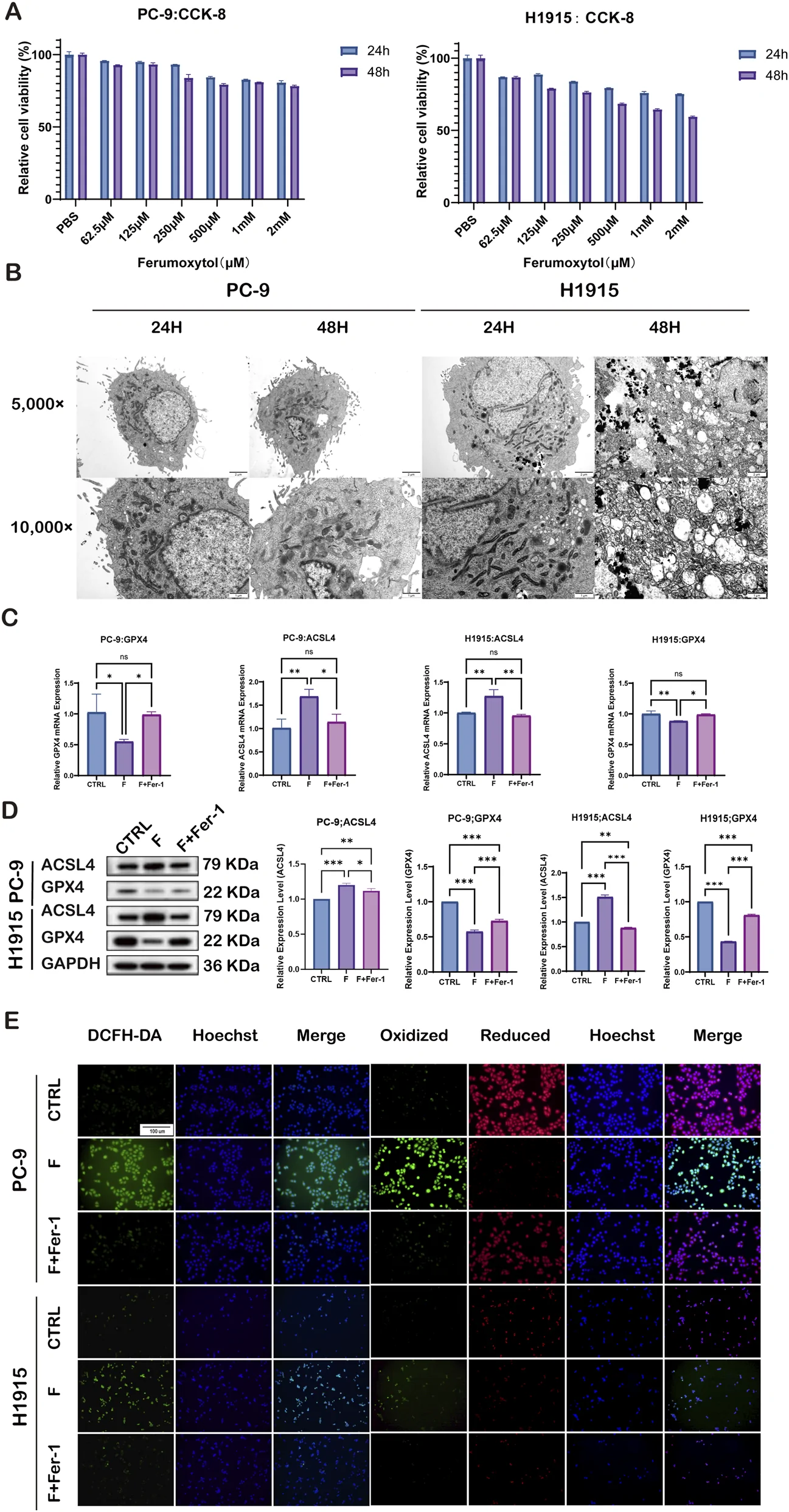
Ferumoxytol induces ferroptosis-related phenotypes in PC-9 and H1915 cells. **(A)** Relative cell viability of PC-9 and H1915 cells after treatment with the indicated concentrations of Ferumoxytol for 24 h or 48 h, as determined by CCK-8 assay (n = 3 per group). **(B)** Representative transmission electron microscopy images of PC-9 and H1915 cells after treatment with Ferumoxytol (250 μM) for 24 h or 48 h, showing ferroptosis-associated mitochondrial alterations, including mitochondrial shrinkage, reduced or lost cristae, and increased membrane density. Images were acquired at ×5,000 and ×10,000 magnification. **(C)** RT-qPCR analysis of GPX4 and ACSL4 mRNA expression in PC-9 and H1915 cells after treatment with Ferumoxytol (250 μM) with or without Ferrostatin-1 (Fer-1, 5 μM) (n = 3 per group). **(D)** Western blot analysis of ACSL4 and GPX4 expression, with densitometric quantification shown on the right. PC-9 and H1915 samples were analyzed on separate blots, and target-protein signals were normalized to the corresponding GAPDH signals from the respective blots for each independent experiment. Representative blots are shown (n = 3 per group). **(E)** shows fluorescence microscopy images of DCFH‐DA staining for ROS and oxidized and reduced C11‐BODIPY staining for lipid peroxidation, together with Hoechst nuclear staining and merged images under the indicated treatment conditions. Scale bar, 100 μm. Data are presented as mean ± SD from three independent experiments. ns, P ≥ 0.05; *P < 0.05; **P < 0.01; ***P < 0.001.

Transmission electron microscopy was used to examine ultrastructural changes following ferumoxytol treatment. At 24 h, both PC-9 and H1915 cells exhibited mitochondrial shrinkage, reduced or absent cristae, and increased membrane density, which are morphological features associated with ferroptosis (Figure 1B). These mitochondrial alterations appeared more prominent in H1915 cells. At 48 h, the ferroptosis-associated mitochondrial changes were less prominent than those observed at 24 h. H1915 cells additionally displayed prominent electron-lucent vacuole-like structures at 48 h. Because these structures were not further characterized using organelle-specific markers or complementary assays, their identity and biological significance remain uncertain.

Because ferroptosis is closely associated with oxidative stress, we further assessed ROS, lipid peroxidation, and intracellular Fe^2+^ levels. After 6 h of Ferumoxytol treatment, ROS levels were increased in both cell lines, accompanied by enhanced lipid peroxidation and stronger Fe^2+^-related fluorescence signals. These changes were attenuated by Fer-1 treatment (Figure 1E; Supplementary Figure S1A; Supplementary Figure S1B). Taken together, Ferumoxytol induced cellular phenotypes, ultrastructural alterations, and molecular marker changes consistent with ferroptosis in both PC-9 and H1915 cells, and these effects were partially reversed by the ferroptosis inhibitor Fer-1.

### 3.2 Ferumoxytol enhances the radiosensitivity of lung cancer cells and brain metastatic lung cancer cells

To further evaluate the effect of Ferumoxytol on the ferroptosis pathway under IR, PC-9 and H1915 cells were assigned to the Ctrl, IR (4 Gy), IR + F (Ferumoxytol, 250 μM), and IR + F + Fer-1 (Fer-1, 5 μM) groups. Cells were collected 24 h after irradiation for Western blot analysis. Compared with the Ctrl group, IR alone altered the expression of ferroptosis-related proteins. Notably, at 24 h after irradiation, IR increased the expression of the ferroptosis-related protein ACSL4 and decreased the expression of the anti-ferroptotic factor GPX4. These changes were more pronounced in the IR + Ferumoxytol group (Figure 2A). In addition, the upregulation of ACSL4 and downregulation of GPX4 were reversed to varying degrees after Fer-1 treatment (Figure 2A). These findings suggest that Ferumoxytol enhances ferroptosis-related molecular changes under irradiation conditions.

**FIGURE 2:**
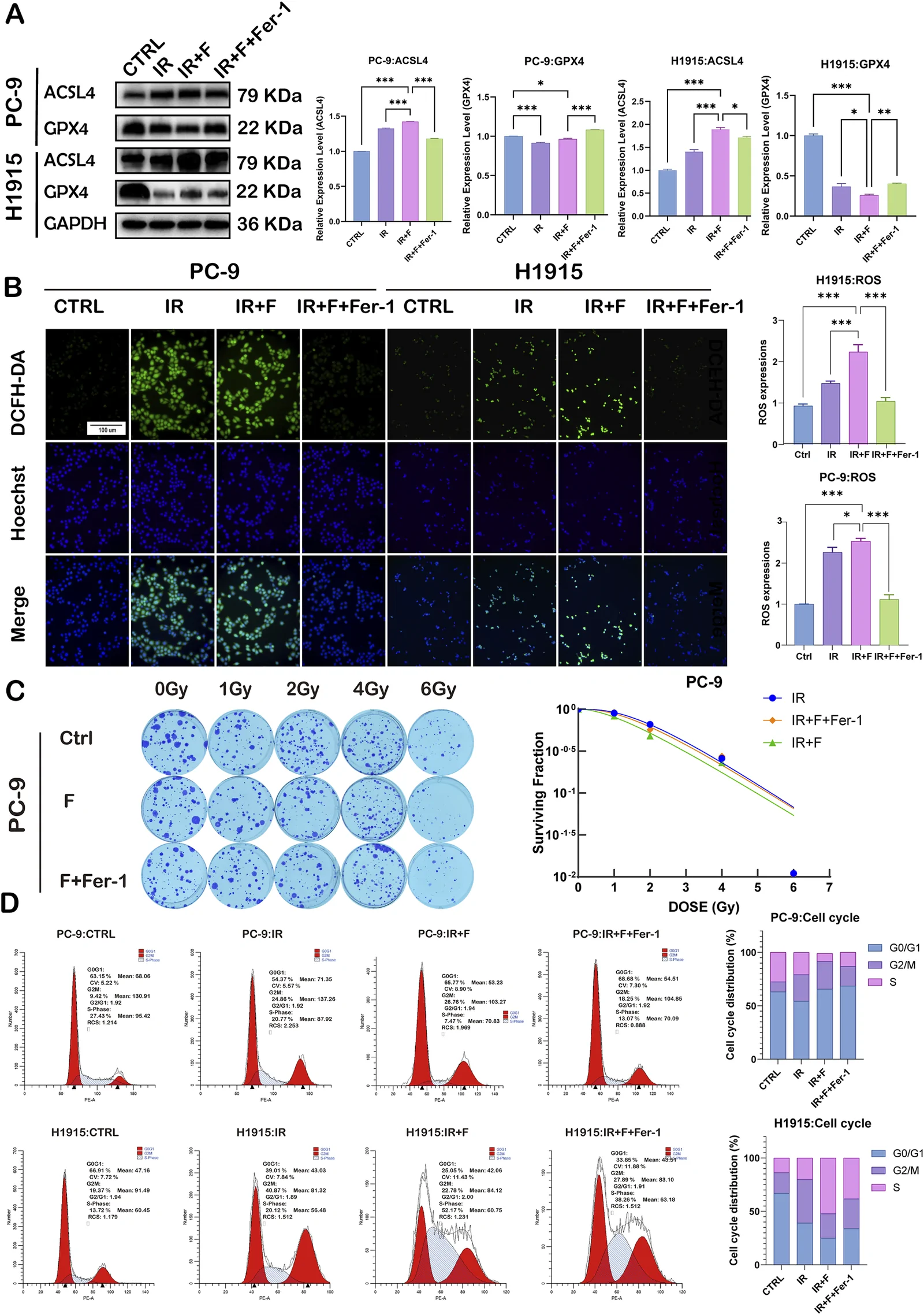
Ferumoxytol enhances ferroptosis-associated oxidative stress and radiosensitivity in PC-9 and H1915 cells. **(A)** PC-9 and H1915 samples were analyzed on separate blots. Target-protein signals were normalized to the corresponding GAPDH signals from the respective blots for each independent experiment. A representative GAPDH strip is shown in the figure montage for display purposes, whereas the statistical analyses were performed using the matched GAPDH signals from the corresponding blots. **(B)** Representative fluorescence images of intracellular ROS detected by DCFH-DA staining at 6 h after the indicated treatments, with quantitative analysis shown on the right. Hoechst was used for nuclear staining. Scale bar, 100 μm. **(C)** Representative clonogenic images of PC-9 cells after the indicated treatments and exposure to 0, 1, 2, 4, or 6 Gy irradiation. Colonies were stained after 14 days, and surviving fractions were fitted using the single-hit multi-target model. The survival curves are shown on the right. **(D)** Representative flow cytometric profiles of cell-cycle distribution in PC-9 and H1915 cells after the indicated treatments, with quantitative analysis of the proportions of cells in G0/G1, S, and G2/M phases shown on the right. Data are presented as mean ± SD from three independent experiments. ns, P ≥ 0.05; *P < 0.05; **P < 0.01; ***P < 0.001.

Because ferroptosis is closely associated with oxidative stress, intracellular ROS and lipid peroxidation were further assessed at 6 h after irradiation. DCFH-DA staining showed that IR increased ROS levels, and the addition of Ferumoxytol further enhanced the ROS signal. This increase was markedly attenuated by Fer-1 (Figure 2B). Similarly, lipid peroxidation was elevated after IR and was further enhanced by the addition of Ferumoxytol (Supplementary Figure S2A). These results suggest that Ferumoxytol amplifies the early oxidative stress response after irradiation, which may facilitate subsequent lipid peroxidation and ferroptotic execution.

We then performed clonogenic assays to assess the effect of Ferumoxytol on radiosensitivity. PC-9 and H1915 cells were exposed to 0, 1, 2, 4, and 6 Gy X-ray irradiation, and dose-survival curves were fitted using the single-hit multi-target model (Figure 2C). Compared with IR alone, combined treatment with Ferumoxytol reduced clonogenic survival in both cell lines, as reflected by a reduced shoulder region of the survival curves and decreased Dq values (Figure 2C; Supplementary Figure S2B). In PC-9 cells, Dq decreased from 1.86 Gy to 1.21 Gy after combined treatment, with a corresponding sensitizer enhancement ratio (SER) of 1.54. In H1915 cells, Dq decreased from 0.50 Gy to 0.32 Gy, with a corresponding SER of 1.56 (Table 1). These results support a radiosensitizing effect of Ferumoxytol.

**TABLE 1:** Clonogenic survival parameters of PC-9 and H1915 cells after the indicated treatments.

Cell line	Group	D0 (Gy)	N	Dq (Gy)	SF_2_	SER*	R^2^
PC-9	IR	1.652	3.083	1.86	0.66	–	0.9939
	IR + F	1.549	2.184	1.21	0.48	1.54	0.9893
	IR + F + Fer-1	1.603	2.817	1.66	0.57	1.12	0.9861
H1915	IR	2.317	1.242	0.50	0.358	–	0.9928
	IR + F	1.645	1.216	0.32	0.309	1.56	0.9906
	IR + F + Fer-1	2.028	1.187	0.35	0.354	1.43	0.9927

Abbreviations: D0, mean lethal dose; N, extrapolation number; Dq, quasi-threshold dose; SF_2_, surviving fraction at 2 Gy; SER, sensitizer enhancement ratio. SER* was calculated using the IR group the reference; SER, Dq (IR)/Dq (IR + treatment).

To further determine whether this sensitizing effect was related to ferroptosis, Fer-1 was added to the combined treatment. Fer-1 partially restored clonogenic survival in both cell lines, increasing Dq to 1.66 Gy in PC-9 cells and 0.35 Gy in H1915 cells, with corresponding SER values of 1.12 and 1.43, respectively (Figure 2C; Table 1). In addition, Fer-1 partially restored the SF2 values. These findings suggest that the radiosensitizing effect of Ferumoxytol is at least partly dependent on ferroptosis.

In addition, to determine whether Ferumoxytol-mediated radiosensitization was associated with cell cycle redistribution, we analyzed the cell cycle profiles of PC-9 and H1915 cells under different treatment conditions by flow cytometry (Figure 2D). IR treatment induced cell cycle remodeling in both cell lines. In PC-9 cells, the proportion of cells in the G2/M phase increased from approximately 9.4% in the control group to approximately 24.9% after IR. In H1915 cells, the G2/M fraction increased from approximately 19.4% to approximately 40.3%, indicating that IR induced G2/M arrest to different extents. Notably, the addition of Ferumoxytol to IR did not further alter the cell cycle distribution pattern in either PC-9 or H1915 cells, and the proportions of cells in each phase remained generally similar to those in the IR group. Likewise, no obvious change in cell cycle distribution was observed after the addition of Ferrostatin-1. These results indicate that although IR induced cell cycle remodeling, the radiosensitizing effect mediated by Ferumoxytol was not dependent on cell cycle redistribution.

### 3.3 Proteomic profiling identifies ferroptosis-related molecular alterations

To further investigate the effect of Ferumoxytol on the molecular profile of H1915 cells under irradiation, we performed proteomic analysis of the IR group (4 Gy) and the IR + F group (4 Gy plus 250 μM ferumoxytol) at 48 h after irradiation. In the data quality assessment, the numbers of identified proteins were similar across samples (R1: 7717, R2: 7566; R + F1: 7630, R + F2: 7537), indicating comparable proteome coverage between groups and supporting the comparability of the dataset (Figure 3A).

**FIGURE 3:**
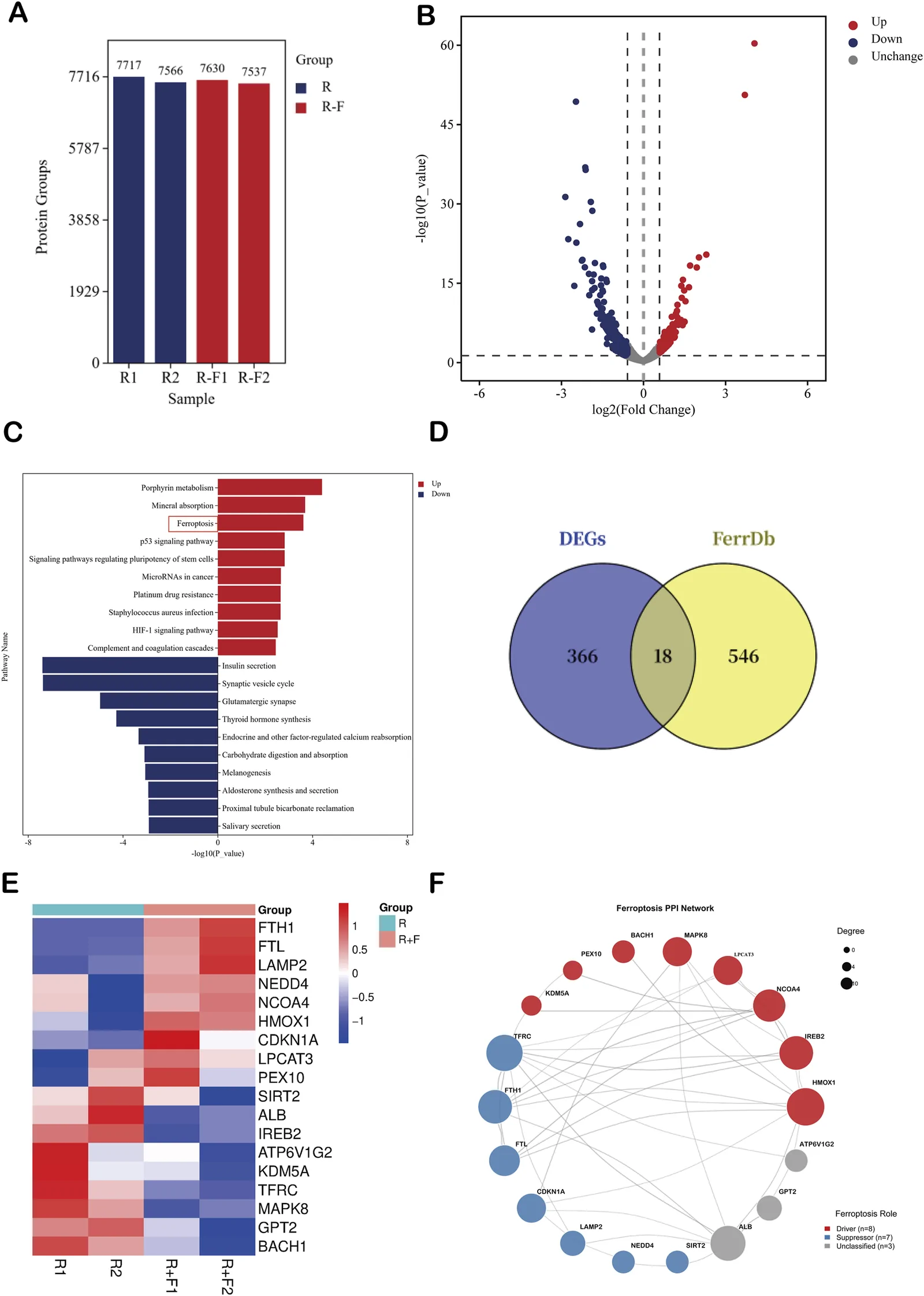
Proteomic analysis reveals ferroptosis-related protein network remodeling in H1915 cells after combined treatment with irradiation and Ferumoxytol. **(A)** Numbers of identified proteins in H1915 cells from the irradiation-alone group (IR, 4 Gy) and the irradiation plus Ferumoxytol group (IR + F, 4 Gy + 250 μM Ferumoxytol). Two biological replicates were included in each group. **(B)** Volcano plot showing differentially expressed proteins in the IR + F group relative to the IR group. Upregulated proteins are shown in red, downregulated proteins in blue, and unchanged proteins in gray. **(C)** KEGG pathway enrichment analysis of differentially expressed proteins between the IR and IR + F groups. Upregulated pathways are shown in red and downregulated pathways in blue. **(D)** Venn diagram showing the overlap between differentially expressed proteins identified by TMT-based proteomics and ferroptosis-related genes from FerrDb. **(E)** Heatmap showing the expression patterns of the 18 overlapping candidate molecules in the IR and IR + F groups. **(F)** Protein-protein interaction network of the 18 ferroptosis-related candidate molecules. Red nodes indicate ferroptosis drivers, blue nodes indicate ferroptosis suppressors, and gray nodes indicate unclassified molecules. Node size represents degree of connectivity. Differentially expressed proteins were defined as those with FC > 1.5 or FC < 0.667 and P < 0.05.

We next compared the protein expression profiles between the two groups. The volcano plot showed a clear set of differentially expressed proteins in the R + F group relative to the R group (Figure 3B). KEGG pathway enrichment analysis of these proteins showed that the upregulated proteins were significantly enriched in porphyrin metabolism, mineral absorption, and ferroptosis, with a prominent enrichment signal for the ferroptosis pathway (Figure 3C). Other enriched upregulated pathways included the p53 signaling pathway and HIF-1 signaling pathway, both of which are associated with stress responses. In contrast, downregulated proteins were enriched in pathways such as insulin secretion and synaptic vesicle cycle (Figure 3C). These findings suggest that the addition of Ferumoxytol under irradiation further reshaped protein networks related to iron metabolism and oxidative stress.

Based on the ferroptosis-related phenotypes observed in the preceding experiments, we further intersected the TMT-derived differentially expressed proteins with the ferroptosis-related gene set from the FerrDb database and identified 18 candidate molecules (Figure 3D). A heatmap was generated to display the expression patterns of these 18 candidates in the R and R + F samples (Figure 3E), including FTH1, FTL, LAMP2, NEDD4, NCOA4, HMOX1, CDKN1A, LPCAT3, PEX10, SIRT2, ALB, IREB2, ATP6V1G2, KDM5A, TFRC, MAPK8, GPT2, and BACH1. Overall, compared with the R group, the R + F group showed expression changes characterized by remodeling of iron homeostasis-related molecules. Notably, the ferritinophagy receptor NCOA4 showed increased expression in the R + F group (Figure 3E).

We then constructed a ferroptosis-related protein-protein interaction (PPI) network based on these 18 candidate molecules. Network analysis showed that these candidates formed a closely connected interaction network and could be classified into ferroptosis drivers, suppressors, and unclassified nodes (drivers, n = 8; suppressors, n = 7; unclassified, n = 3). Among them, NCOA4, identified as one of the driver nodes, occupied a key connecting position within the network (Figure 3F). Based on its altered expression, central network position, and established role in ferritinophagy, NCOA4 was prioritized for further evaluation. We next examined whether public single-cell and spatial transcriptomic datasets of lung cancer brain metastasis also supported metastasis-associated remodeling of ferroptosis-related genes and an NCOA4-centered ferroptosis program.

### 3.4 Public single-cell analyses support metastasis-associated remodeling of ferroptosis-related genes

To further determine whether the ferroptosis-associated genes highlighted by our experimental and proteomic analyses were also supported in public transcriptomic datasets, we next analyzed a public single-cell dataset of lung cancer brain metastasis. Integrated single-cell annotation identified multiple cellular compartments, including tumor cells, myeloid-lineage populations, microglia, endothelial cells, fibroblasts, and lymphoid cells, indicating that brain metastatic lesions were embedded in a complex multicellular microenvironment (Figure 4A). When samples were grouped by origin, brain metastasis and primary tumor cells showed partially distinct distribution patterns across the integrated atlas (Figure 4B), suggesting metastasis-associated transcriptional remodeling at the tissue level.

**FIGURE 4:**
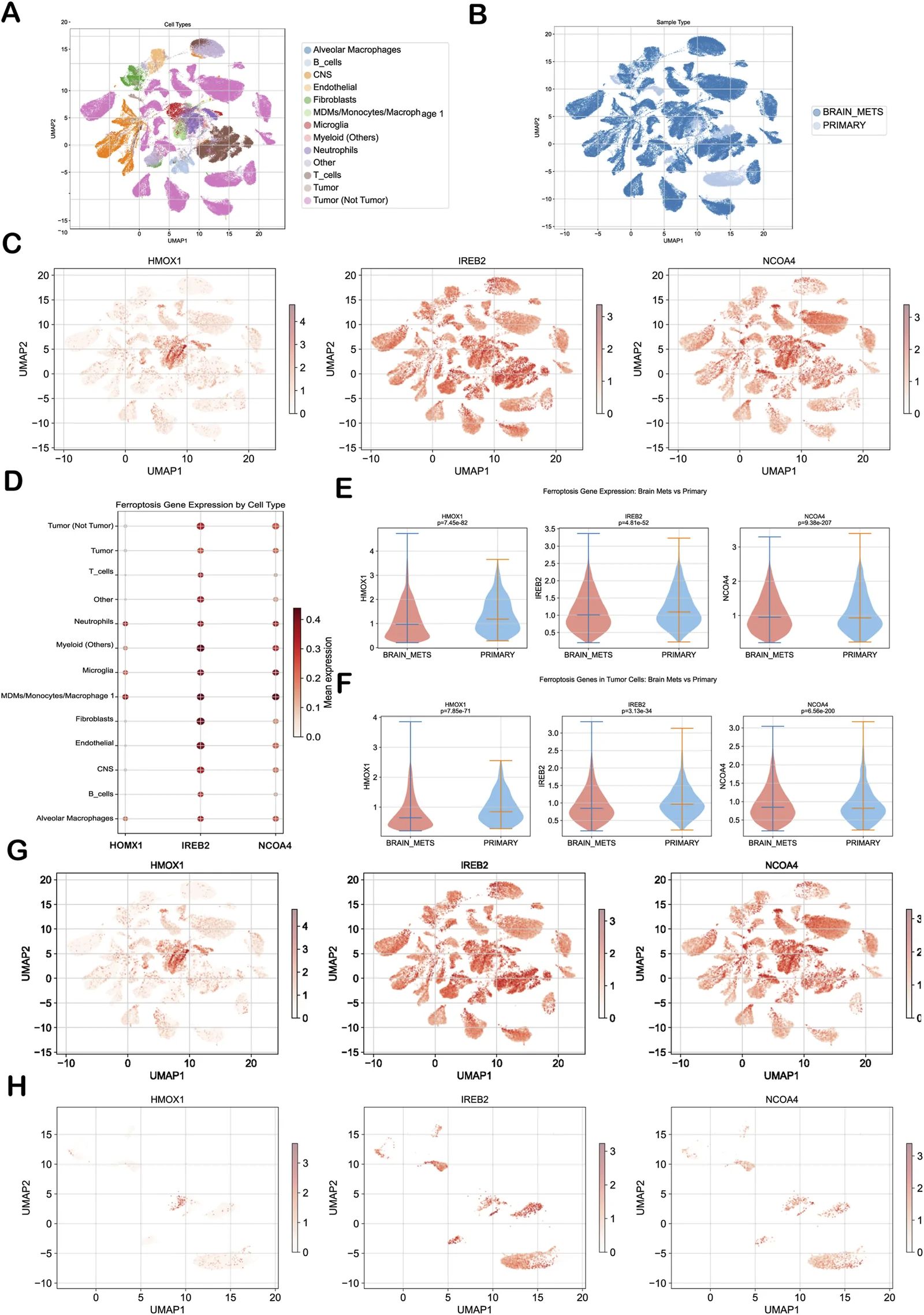
Public single-cell analyses support metastasis-associated remodeling of ferroptosis-related genes in lung cancer brain metastasis. **(A)** UMAP visualization of the integrated single-cell atlas colored by annotated cell type. **(B)** UMAP visualization colored by sample origin, showing the distribution of primary tumors and brain metastases in the integrated atlas. **(C)** UMAP feature plots showing the expression of HMOX1, IREB2, and NCOA4 in the integrated dataset. **(D)** Dot plot showing the expression level and detection frequency of HMOX1, IREB2, and NCOA4 across major cell populations. **(E)** Violin plots comparing the expression of HMOX1, IREB2, and NCOA4 between primary tumors and brain metastases across the whole atlas. **(F)** Violin plots comparing the expression of HMOX1, IREB2, and NCOA4 between primary tumors and brain metastases within the tumor-cell compartment. **(G)** Feature plots showing the expression of HMOX1, IREB2, and NCOA4 in the brain-metastasis subset. **(H)** Feature plots showing the expression of HMOX1, IREB2, and NCOA4 in the primary-tumor subset.

We then examined the expression patterns of HMOX1, IREB2, and NCOA4 in the integrated atlas. Feature plots and cell type-resolved dot plots showed that these ferroptosis-associated genes displayed heterogeneous and partially cell type-specific expression patterns rather than uniform distribution across all compartments (Figures 4C,D). In particular, NCOA4 was detectable in tumor-related compartments and remained interpretable in the broader brain metastatic microenvironment, supporting its relevance beyond the *in vitro* system. These findings indicate that the genes implicated by our wet-lab experiments are embedded within a biologically structured metastatic ecosystem rather than representing isolated cell line phenomena.

To further evaluate whether these differences were associated with metastatic status, we compared gene expression between primary and brain metastatic samples at both the whole-atlas level and within the tumor compartment. Violin plot analysis showed metastasis-associated differences in HMOX1, IREB2, and NCOA4 across the integrated dataset (Figure 4E). Importantly, these differences were still detectable when the analysis was restricted to tumor cells (Figure 4F), arguing against a purely compositional explanation based on shifts in cell-type abundance alone. Additional feature visualization of the brain metastasis and primary subsets further supported distinct expression contexts for these genes between the two groups (Figures 4G,H). Collectively, these public single-cell data support metastasis-associated remodeling of ferroptosis-related genes and provide an external expression context for the prioritization of NCOA4 from our proteomic network analysis.

### 3.5 Spatial transcriptomic and expression-stratified *in silico* analyses identify an NCOA4-associated ferroptosis-defense signature in brain metastasis

Because brain metastatic lesions are spatially heterogeneous and shaped by complex tumor–microenvironment interactions, we next investigated whether the ferroptosis-related genes prioritized by our proteomic analysis exhibited spatial organization in public lung cancer brain metastasis transcriptomic data. Spatial feature mapping in a representative brain metastatic lesion showed that HMOX1, IREB2, and NCOA4 were not uniformly distributed across the tissue section but displayed distinct lesion-level expression patterns (Figure 5A). HMOX1 showed relatively broad and regionally enriched expression, whereas IREB2 and NCOA4 displayed more heterogeneous and spatially restricted signals. These findings suggest that ferroptosis-associated transcriptional states are spatially organized within brain metastatic lesions rather than being evenly distributed across the tumor tissue.

**FIGURE 5:**
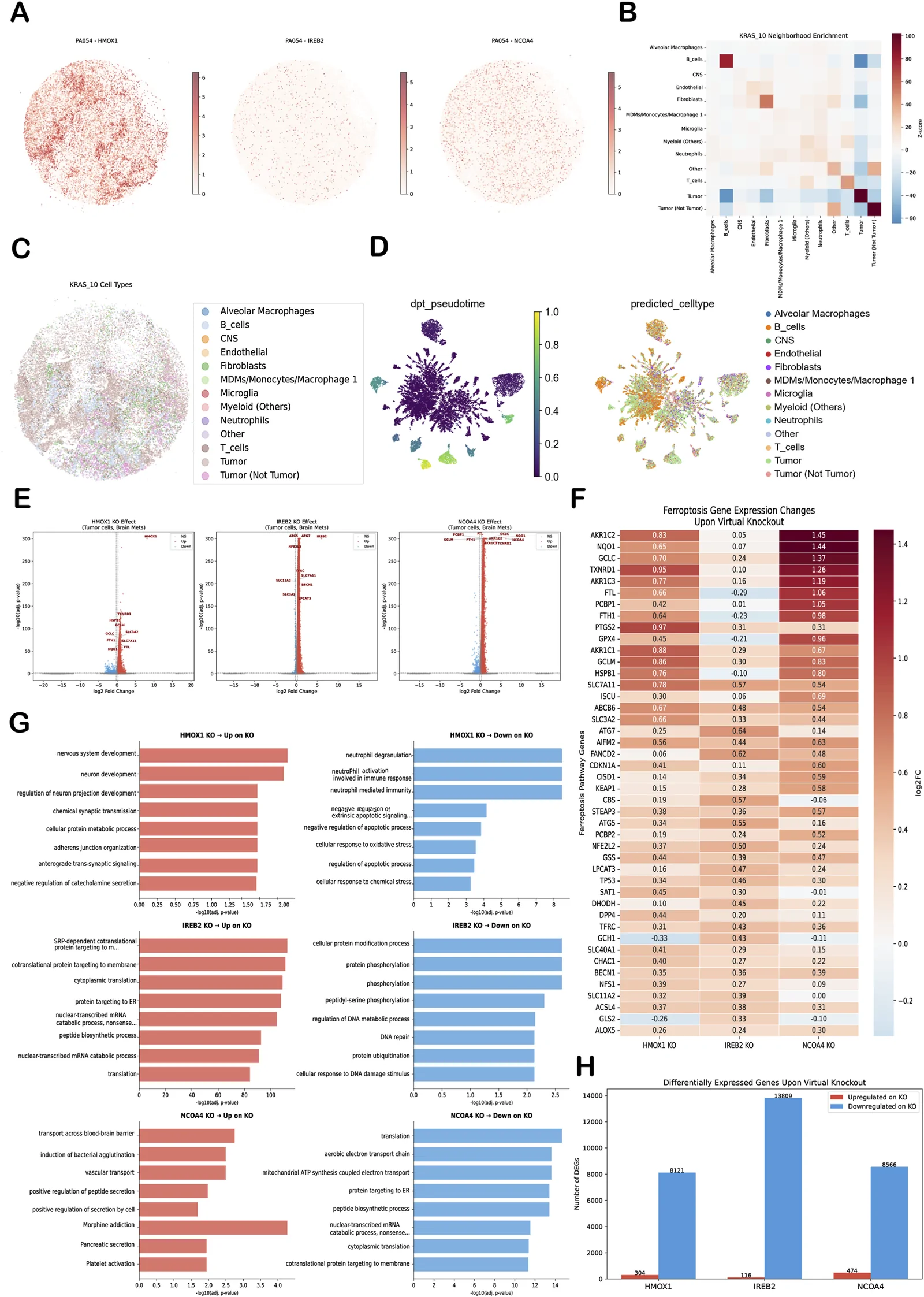
Spatial and expression-stratified *in silico* analyses support an NCOA4-associated ferroptosis program in brain metastasis. **(A)** Representative spatial feature plots showing lesion-level HMOX1, IREB2, and NCOA4 expression. **(B)** Neighborhood-enrichment analysis of predicted cell populations. **(C)** Representative spatial cell-type map. **(D)** Trajectory-like analysis illustrating a continuous transcriptional-state spectrum; this analysis is not interpreted as a lineage reconstruction. **(E)** Volcano plot comparing NCOA4 expression strata in the brain-metastasis tumor-cell subset. **(F)** Ferroptosis-focused heatmap showing genes associated with the NCOA4 expression strata. **(G)** Functional enrichment of genes differing between NCOA4 strata. **(H)** Numbers of differentially expressed genes associated with HMOX1, IREB2, and NCOA4 expression strata. These computational analyses are associative and do not represent genetic knockout experiments.

To further define the cellular context in which these spatial signals emerged, we performed spatial cell-type mapping and neighborhood enrichment analysis. The representative lesion contained multiple predicted cellular compartments, including tumor-related cells, myeloid-lineage populations, microglia, endothelial cells, fibroblasts, lymphoid cells, and other stromal or CNS-related populations (Figures 5B,C). Neighborhood enrichment analysis further revealed non-random spatial relationships among these cell populations, supporting the presence of a structured metastatic ecosystem rather than a homogeneous tumor mass (Figure 5B). These observations indicate that ferroptosis-related gene expression in brain metastasis occurs within a spatially organized tumor microenvironment, where tumor cells and microenvironmental populations may jointly shape iron metabolism- and oxidative stress-related states.

We next evaluated whether the spatially resolved metastatic lesion also showed continuous transcriptional state organization. Diffusion pseudotime analysis revealed a spectrum of transcriptional states across the lesion, and these states were accompanied by heterogeneous predicted cell-type composition (Figure 5D). Although this trajectory-like analysis should not be interpreted as a direct lineage relationship, it suggests that brain metastatic lesions contain gradual state transitions rather than completely discrete cellular states. This finding provides additional context for the observed spatial heterogeneity of HMOX1, IREB2, and NCOA4 and supports the concept that ferroptosis-related vulnerability may be state-dependent within brain metastases.

To explore the transcriptional context associated with these ferroptosis-related genes, we used the expression-stratified *in silico* analysis described in the Methods. This comparison did not constitute a genetic virtual knockout. Within the brain-metastasis tumor-cell subset, NCOA4-low/negative cells differed broadly from NCOA4-high/positive cells (Figure 5E). The NCOA4-low/negative state was associated with increased expression of antioxidant-defense, glutathione-metabolism, and iron-sequestration genes, including NQO1, TXNRD1, GCLC/GCLM, GPX4, FTH1, FTL, and PCBP1 (Figure 5F). These associations are consistent with a more iron-buffered transcriptional state but cannot establish that loss of NCOA4 caused the observed changes.

Pathway enrichment analysis of genes differing between the NCOA4 expression strata showed enrichment of transport- and secretion-related processes among genes higher in the NCOA4-low/negative state, whereas mitochondrial electron transport, ATP synthesis, translation, and peptide-biosynthetic programs were lower (Figure 5G). Comparisons for HMOX1, IREB2, and NCOA4 yielded substantial expression-associated gene sets (Figure 5H). NCOA4 remained the most relevant candidate for experimental follow-up because it was independently prioritized by TMT proteomics and the ferroptosis-related protein-interaction network; the computational results themselves were treated as associative.

Together, the public single-cell and spatial analyses provide external disease-context support for an NCOA4-associated ferroptosis program in lung cancer brain metastasis. The association between lower NCOA4 expression and antioxidant/iron-sequestration signatures is directionally consistent with the *in vitro* observation that NCOA4 silencing attenuated combined-treatment Fe^2+^ accumulation, oxidative stress, and γ-H2AX induction. Nevertheless, only the siRNA experiments provide direct loss-of-function evidence in this study.

### 3.6 NCOA4 contributes to ferumoxytol-enhanced oxidative damage and the incremental clonogenic response under irradiation

Given the convergent candidate-prioritization results, we performed direct loss-of-function experiments. In H1915 cells, siNCOA4 reduced NCOA4 protein and mRNA expression by approximately 80% relative to NC (Figure 6A). Independent immunoblotting also confirmed NCOA4 reduction in PC-9 cells (Supplementary Figure S3A). These experiments established effective knockdown in both the brain-metastatic H1915 model and a non-brain-metastatic NSCLC comparison model.

**FIGURE 6:**
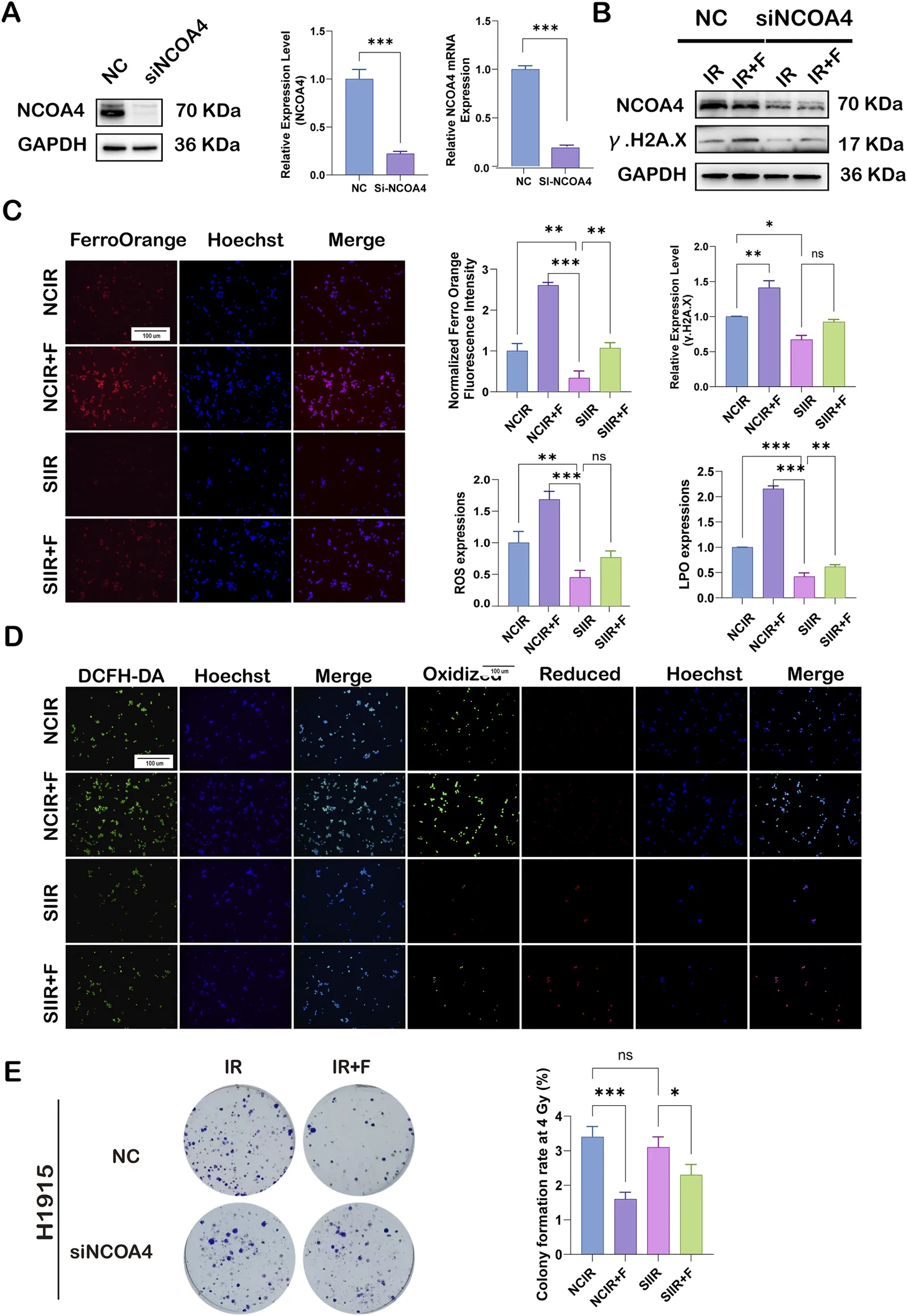
NCOA4 contributes to ferumoxytol-enhanced oxidative damage and the incremental clonogenic response under irradiation in H1915 cells. **(A)** NCOA4 knockdown validated by Western blot and RT-qPCR (n = 3). **(B)** NCOA4 and γ-H2AX immunoblotting 2 h after 8 Gy IR with or without ferumoxytol in NC- and siNCOA4-transfected cells; the graph shows γ-H2AX/GAPDH (n = 3). **(C)** FerroOrange imaging and quantification of labile Fe^2+^. Hoechst was used for nuclear staining; scale bar, 100 μm. **(D)** DCFH-DA imaging of ROS and C11-BODIPY 581/591 imaging of lipid peroxidation, with quantitative analyses. Hoechst was used for nuclear staining; scale bar, 100 μm. **(E)** Representative 4-Gy single-dose colony-formation assay after NC or siNCOA4 transfection, with colony formation rate (%) quantified on the right (1,000 cells/well; n = 3). Data are mean ± SD. ns, P ≥ 0.05; *P < 0.05; **P < 0.01; ***P < 0.001.

We next assessed early DNA-damage signaling. H1915 cells with NC or siNCOA4 were exposed to 8 Gy and collected 2 h later. Under NC conditions, γ-H2AX was higher after IR + F than after IR alone. Under siNCOA4 conditions, γ-H2AX abundance was lower overall, and the IR + F versus IR difference was not significant (Figure 6B). PC-9 cells showed the same directional reduction in γ-H2AX after NCOA4 knockdown, and no significant difference was detected between siNCOA4+IR and siNCOA4+IR + F (Supplementary Figure S3B). These findings support a contribution of NCOA4 to the early combined-treatment damage phenotype but do not identify γ-H2AX as a ferroptosis-specific endpoint.

We then assessed labile Fe^2+^, ROS, and lipid peroxidation in H1915 cells. In the NC background, IR + F increased FerroOrange, DCFH-DA, and oxidized C11-BODIPY signals relative to IR. NCOA4 silencing lowered these signals and attenuated the increment produced by adding ferumoxytol (Figures 6C,D). These results place NCOA4 upstream of part of the iron-associated oxidative phenotype, while the residual response indicates that NCOA4 is not the sole determinant.

To determine whether NCOA4 affected the incremental clonogenic response to ferumoxytol, we performed 4-Gy single-dose colony-formation assays. In NC-transfected H1915 and PC-9 cells, adding ferumoxytol to IR significantly reduced colony formation. Under NCOA4 knockdown, this incremental reduction was smaller: it remained modestly significant in H1915 cells but was not significant in PC-9 cells (Figure 6E; Supplementary Figure S3C). Because siNCOA4 also altered colony formation after IR alone, particularly in PC-9 cells, these data are interpreted as attenuation of the incremental ferumoxytol effect rather than as uniform radioprotection by NCOA4 loss.

### 3.7 IR + F alters NCOA4-FTH1 dynamics and engages an autophagy-associated ferritin-handling response

We first examined NCOA4 and FTH1 over time in H1915 cells after IR + F. NCOA4 remained close to the control level from 6 to 24 h and decreased at 48 h. In contrast, FTH1 increased markedly at every measured time point and was highest at 48 h (Figure 7A). Thus, the time course did not demonstrate net FTH1 depletion. Instead, it indicates a prominent ferritin-accumulation response, which may reflect compensatory ferritin synthesis and iron sequestration during sustained exogenous iron loading and oxidative stress.

**FIGURE 7:**
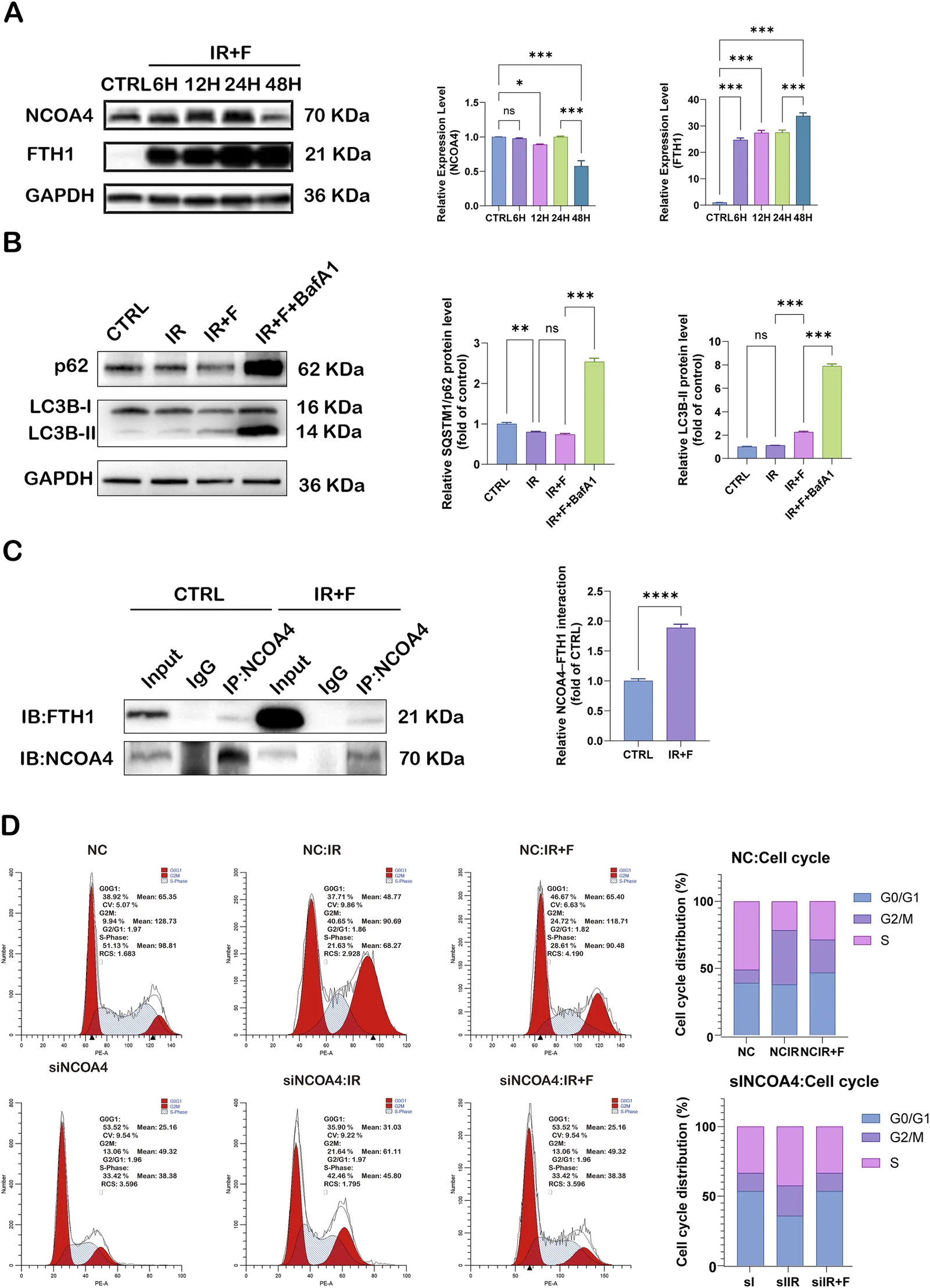
IR + F alters NCOA4-FTH1 dynamics and engages an autophagy-associated ferritin-handling response in H1915 cells. **(A)** Time-resolved immunoblotting of NCOA4 and FTH1 at 0, 6, 12, 24, and 48 h after IR + F, with GAPDH-normalized quantification (n = 3). **(B)** SQSTM1/p62 and LC3B-I/II immunoblotting in Ctrl, IR, IR + F, and IR + F + BafA1 groups; Bafilomycin A1 (BafA1, 0.6 nM) was added during the final 4 h before sample collection, and cells were collected 24 h after irradiation. Quantification shows SQSTM1/p62/GAPDH and LC3B-II/GAPDH (n = 3). **(C)** Endogenous NCOA4-FTH1 co-immunoprecipitation in Ctrl and IR + F groups. Input and IgG controls are shown. For quantification, co-immunoprecipitated FTH1 was normalized to immunoprecipitated NCOA4, and the control value was set to 1 (n = 3). **(D)** Representative cell-cycle profiles of H1915 cells transfected with NC or siNCOA4 under untreated, IR, and IR + F conditions, with phase distributions summarized on the right (n = 3). Data are mean ± SD where error bars are shown. ns, P ≥ 0.05; *P < 0.05; **P < 0.01; ***P < 0.001; ****P < 0.0001.

We next evaluated autophagic flux. Relative to IR + F alone, addition of BafA1 produced marked accumulation of LC3B-II and SQSTM1/p62 (Figure 7B), consistent with ongoing autophagic turnover that becomes detectable when lysosomal degradation is blocked. This result supports engagement of the autophagy-lysosome system during combined treatment, but it is not by itself specific for ferritin cargo.

Endogenous co-immunoprecipitation recovered FTH1 in NCOA4 immunoprecipitates. After normalization of co-immunoprecipitated FTH1 to immunoprecipitated NCOA4, the relative FTH1 recovery was approximately 1.9-fold higher in the IR + F group than in the Ctrl group (Figure 7C). The IgG control was included to assess nonspecific binding. Because cellular FTH1 abundance also increased after IR + F, this result supports treatment-associated recovery of the NCOA4–FTH1 complex but does not distinguish altered interaction propensity from increased FTH1 availability, nor does it establish lysosomal delivery, ferritin degradation, or iron release.

Finally, cell-cycle profiling showed that IR remodeled cell-cycle distribution in both NC and siNCOA4 backgrounds, whereas adding ferumoxytol produced no clear additional redistribution (Figure 7D). NCOA4 knockdown altered the baseline and irradiation-associated cell-cycle patterns, but the lack of an additional IR + F effect argues against cell-cycle redistribution as the principal explanation for ferumoxytol-mediated radiosensitization.

## 4 Discussion

Brain metastasis is a major cause of death and neurological dysfunction in patients with advanced NSCLC (Sun et al., 2024). Although radiotherapy remains a principal local treatment, the tolerance of normal brain tissue limits dose escalation (Tanzhu et al., 2025). Because IR generates ROS and perturbs iron homeostasis (Dixon et al., 2012; Lei et al., 2021; Li et al., 2021; Zou et al., 2021), we examined whether ferumoxytol could amplify iron-associated oxidative injury. The results support enhanced lipid peroxidation and loss of clonogenic survival and implicate NCOA4-associated ferritin handling as one contributor. They do not establish direct ferritin-derived iron release or *in vivo* radiosensitization.

The present study first showed that ferumoxytol alone induced ferroptosis-associated phenotypes in PC-9 and H1915 cells, including ROS accumulation, increased labile Fe^2+^, lipid peroxidation, and disruption of the GPX4/ACSL4 axis. Previous work indicates that intracellular processing of ferumoxytol can increase the availability of redox-active iron, which catalyzes H_2_O_2_-dependent Fenton chemistry under appropriate microenvironmental conditions (Nolte et al., 2013; Petronek et al., 2023; Tanzhu et al., 2025). The bidirectional redox behavior reported across cellular systems reinforces the need for context-specific mechanistic and *in vivo* safety validation.

More importantly, ferumoxytol further enhanced several irradiation-associated ferroptosis-related effects. Clonogenic assays showed that IR + F reduced the surviving fraction compared with IR alone, while ROS accumulation and lipid peroxidation were further increased. These findings suggest that ferumoxytol may enhance radiation-associated injury by amplifying oxidative stress initiated by IR. Ferrostatin-1 only partially restored clonogenic survival, indicating that ferroptosis-associated injury contributes to, but does not fully account for, the cytotoxicity of the combined treatment; direct radiation-induced DNA damage and other cell-death pathways likely remain operative. Cell-cycle analysis showed no clear additional redistribution after ferumoxytol was added to IR, arguing against cell-cycle redistribution as the principal explanation for the observed radiosensitizing effect. In light of evidence that irradiation may promote oxidative dissolution of iron-containing nanoparticles and increase labile iron availability (Petronek et al., 2023), we speculate that IR increases the initial oxidative burden, including endogenous H_2_O_2_, whereas intracellular processing of ferumoxytol increases the availability of redox-active iron that catalyzes Fenton chemistry. Together, these processes may amplify lipid peroxidation and ferroptosis-associated oxidative injury.

At the mechanistic level, the present data implicate NCOA4 as a contributor to the radiation-associated ferroptosis phenotype. NCOA4 is a cargo receptor that binds FTH1 and participates in ferritin delivery to the autophagy–lysosome system (Wang et al., 2023; Wu et al., 2023; Chen et al., 2026). In this study, NCOA4 was independently prioritized by TMT proteomics and a ferroptosis-related protein-interaction network. NCOA4 silencing attenuated Fe^2+^ accumulation, ROS production, lipid peroxidation, γ-H2AX induction, and the incremental clonogenic effect of adding ferumoxytol to IR. BafA1-based analysis showed ongoing autophagic turnover under IR + F, and endogenous co-immunoprecipitation confirmed the recovery of FTH1 in NCOA4 immunoprecipitates, with greater normalized FTH1 recovery after IR + F. Because cellular FTH1 abundance also increased after treatment, the co-immunoprecipitation result does not by itself establish increased binding affinity, lysosomal cargo delivery, or ferritin degradation. Together, these findings support NCOA4-associated ferritin handling as one contributor to the combined-treatment phenotype (Mancias et al., 2014).

The expression-stratified *in silico* analysis provides an additional, associative layer of support. Lower NCOA4 expression was associated with antioxidant and iron-sequestration signatures, which is directionally consistent with the siRNA phenotype. This computational concordance should not be interpreted as causal proof: the groups were defined by observed expression rather than by an experimentally imposed knockout. The analysis therefore places the experimentally identified NCOA4 phenotype in a disease-relevant transcriptional context without replacing direct functional validation. Recent experimental work in other systems also links irradiation, NCOA4-associated ferritinophagy, iron release, and ferroptosis-related injury (Qian et al., 2026), but whether the same complete sequence operates here remains to be established.

Based on these findings, we propose a deliberately limited working model. Ferumoxytol provides an exogenous iron load, while IR supplies an initial ROS and membrane-oxidation pressure. NCOA4-associated engagement with ferritin may contribute to intracellular ferritin handling and the availability of redox-active iron, thereby amplifying lipid peroxidation and ferroptosis-associated damage. Importantly, FTH1 accumulated rather than showing net depletion during the measured time course. Although BafA1-dependent LC3B-II/SQSTM1 accumulation and NCOA4-FTH1 co-immunoprecipitation strengthen the mechanistic link, they do not demonstrate cargo delivery to autolysosomes or net ferritin degradation. We therefore use the term “NCOA4-associated iron mobilization” and do not claim that a complete canonical ferritinophagy sequence has been established.

From a conceptual perspective, the efficacy of radiotherapy may depend not only on DNA double-strand breaks but also on whether oxidative injury can be amplified in an iron-vulnerable cellular state. The present data support an association among exogenous iron loading, NCOA4-dependent ferritin handling, ferroptosis-associated oxidative injury, and radiosensitivity. They do not establish that ferritinophagy is the exclusive mechanism, and the partial Fer-1 rescue and residual phenotypes after NCOA4 knockdown indicate that parallel radiation-damage pathways remain operative.

From a translational perspective, the appeal of Ferumoxytol lies not only in its potential radiosensitizing effect, but also in its value for drug repurposing and imaging translation. Ferumoxytol was originally developed as a clinical iron supplement and has subsequently been extended to tumor MRI and theranostic applications. Previous studies have shown that as a brain MRI contrast agent, Ferumoxytol can effectively delineate malignant brain tumor lesions associated with blood-brain barrier disruption, suggesting substantial progress in its clinical imaging translation in brain tumor settings (Iv et al., 2019; Petronek et al., 2024; Yagmurlu et al., 2024). In addition, Chang et al. found in a bilateral breast tumor radiotherapy model that although Ferumoxytol did not enhance the abscopal effect, MRI revealed changes in its uptake in tumors and lymph nodes, highlighting its value for monitoring the tumor microenvironment (Chang et al., 2025). Therefore, in the setting of lung cancer brain metastasis, the future potential of Ferumoxytol may extend beyond radiosensitization alone and may include integrated applications in drug distribution assessment, lesion imaging, and treatment monitoring.

Several limitations should be emphasized. First, all mechanistic experiments were performed *in vitro*. These models cannot reproduce ferumoxytol pharmacokinetics, blood-brain barrier disruption, intracranial distribution, tumor-microenvironment interactions, fractionated-radiotherapy scheduling, or normal-brain toxicity (Maheshwari et al., 2025; Rhee et al., 2025). No claim of *in vivo* efficacy or clinical radiosensitization can therefore be made. Second, the main proteomic screen and the most extensive NCOA4 functional analyses were performed in H1915 cells. PC-9 validation improves cross-model support but does not substitute for additional brain-metastatic models. Moreover, the proteomic comparison included two biological replicates per group and should be regarded as an exploratory prioritization step rather than definitive quantitative evidence.

Third, the BafA1-based autophagic-turnover and NCOA4–FTH1 co-immunoprecipitation experiments strengthen the proposed mechanistic association but do not fully establish canonical ferritinophagy. FTH1 accumulated during the measured IR + F time course, and lysosomal colocalization, cargo-specific tandem-reporter assays, ferritin half-life measurements, and direct demonstration of ferritin-derived iron release were not performed. Iron-chelator rescue was also not completed, limiting discrimination between redox-active iron dependence and other oxidative mechanisms. In addition, the prominent electron-lucent vacuole-like structures observed in H1915 cells at 48 h were not further characterized and therefore cannot be assigned to autophagic, lysosomal, or ferroptosis-specific compartments on morphology alone. Fourth, Fer-1 was not tested within the siNCOA4 background. The Fer-1 rescue and NCOA4 loss-of-function experiments should therefore be interpreted as complementary rather than as a formal epistasis test. Finally, the public transcriptomic analyses are associative. Their expression-stratified comparisons provide disease-context support but cannot replace experimental perturbation.

In summary, ferumoxytol increased radiation-associated oxidative stress, lipid peroxidation, and loss of clonogenic survival in the tested lung cancer cell models. NCOA4 loss of function attenuated Fe^2+^, oxidative, DNA-damage, and incremental clonogenic responses. Autophagic-flux and co-immunoprecipitation experiments further support treatment-associated engagement of an NCOA4-FTH1 ferritin-handling pathway, while the FTH1 time course argues against claiming simple net ferritin degradation. These findings warrant *in vivo* validation and more direct cargo-specific studies before translational conclusions are drawn (Figure 8).

**FIGURE 8:**
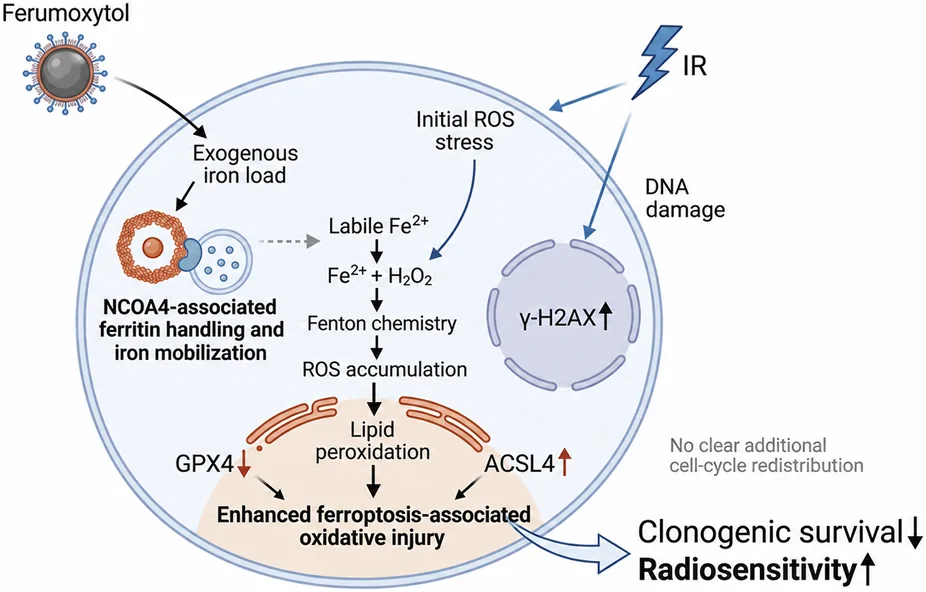
Proposed working model of ferumoxytol-enhanced radiation-associated oxidative injury in lung cancer cells. Following intracellular processing, ferumoxytol increases the availability of redox-active iron, while ionizing radiation initiates oxidative stress and DNA damage. NCOA4-associated ferritin handling may contribute to labile Fe^2+^ availability, thereby amplifying Fenton chemistry, ROS accumulation, lipid peroxidation, and ferroptosis-associated oxidative injury. These changes are accompanied by increased γ-H2AX, reduced clonogenic survival, and increased radiosensitivity. NCOA4 silencing partially attenuated these responses. The schematic summarizes the *in vitro* findings and does not represent an *in vivo* or intracranial model.

## 5 Conclusion

This study shows that ferumoxytol enhances irradiation-associated oxidative damage, lipid peroxidation, and loss of clonogenic survival in lung cancer cell models. NCOA4 knockdown, BafA1-based flux analysis, and NCOA4-FTH1 co-immunoprecipitation support the involvement of NCOA4-associated ferritin handling in this response. Because FTH1 accumulated rather than undergoing net depletion and because iron-chelator, cargo-specific lysosomal, and *in vivo* validation were not performed, the data should be interpreted as evidence for an NCOA4-associated mechanism rather than definitive proof of canonical ferritinophagy or clinical efficacy.

## Data Availability

The datasets presented in this study can be found in online repositories. The names of the repository/repositories and accession number(s) can be found in the article/Supplementary Material.
