# Supplementary material for: Ferumoxytol potentiates ferroptotic radiosensitization through NCOA4-associated iron mobilization in brain-metastatic lung cancer cells
Source: Front Pharmacol. 2026 Oct 14;17:1883092. doi: 10.3389/fphar.2026.1883092 (PMC13713803; doi:10.3389/fphar.2026.1883092)
Supplement: Supplementary file 1 [file Supplementaryfile2.docx]

***Supplementary Table***

**Supplementary Table 1. Antibodies used in this study**

| **Antibody** | **Supplier** | **Catalog no.** | **Host / clonality** | **Application** | **Working dilution** |
| --- | --- | --- | --- | --- | --- |
| **GPX4** | Abcam | ab125066 | Rabbit monoclonal | WB | 1:1000 |
| **γ-H2AX (pS139)** | Abcam | ab81299 | Rabbit monoclonal | WB | 1:1000 |
| **NCOA4** | Abcam | ab86707 | Rabbit polyclonal | WB | 1:1000 |
| **ACSL4** | Proteintech | 22401-1-AP | Rabbit polyclonal | WB | 1:3000 |
| **GAPDH** | Proteintech | 60004-1-Ig | Mouse monoclonal | WB | 1:5000 |
| **Goat anti-rabbit IgG-HRP** | Abcam | ab6721 | Goat | WB | 1:5000 |
| **Goat anti-mouse IgG-HRP** | Abcam | ab6789 | Goat | WB | 1:5000 |

**Supplementary Table 2. siRNA sequences used in this study**

| **Name** | **Sense (5′–3′)** | **Antisense (5′–3′)** | **Supplier** | **Application** |
| --- | --- | --- | --- | --- |
| **siNCOA4 (NCOA4-Homo-1656)** | GCAUAAAGAUUCCCUGAAUTT | AUUCAGGGAAUCUUUAUGCTT | GenePharma (Shanghai, China) | Knockdown |
| **Negative control siRNA** | UUCUCCGAACGUGUCACGUTT | ACGUGACACGUUCGGAGAATT | GenePharma (Shanghai, China) | Negative control |

**Supplementary Table 3. Primer sequences used for qRT-PCR**

| **Gene** | **RefSeq accession** | **Forward primer (5′–3′)** | **Reverse primer (5′–3′)** | **Supplier** | **Application** |
| --- | --- | --- | --- | --- | --- |
| **GPX4** | NM_002085 | GAGGCAAGACCGAAGTAAACTAC | CCGAACTGGTTACACGGGAA | GenePharma (Shanghai, China) | qRT-PCR |
| **ACSL4** | NM_004458 | CATCCCTGGAGCAGATACTCT | TCACTTAGGATTTCCCTGGTCC | GenePharma (Shanghai, China) | qRT-PCR |
| **NCOA4** | NM_005437 | ATGGCAGAAGCATTGAAAGC | TTGGCTTCTGTAAACGTGAGG | GenePharma (Shanghai, China) | qRT-PCR |
| **GAPDH** | NM_002046 | GGAGCGAGATCCCTCCAAAAT | GGCTGTTGTCATACTTCTCATGG | GenePharma (Shanghai, China) | qRT-PCR |
