# Supplementary material for: Ferumoxytol potentiates ferroptotic radiosensitization through NCOA4-associated iron mobilization in brain-metastatic lung cancer cells
Source: Front Pharmacol. 2026 Oct 14;17:1883092. doi: 10.3389/fphar.2026.1883092 (PMC13713803; doi:10.3389/fphar.2026.1883092)
Supplement: Supplementary file 2 [file Supplementaryfile3.docx]

***Supplementary Figures***

**Fig. S1. Fer-1 partially attenuates Ferumoxytol-induced oxidative stress, lipid peroxidation, and Fe²⁺ accumulation in PC-9 and H1915 cells.** **A.** Quantitative analysis of intracellular ROS and lipid peroxidation (LPO) in PC-9 and H1915 cells after 6 h of treatment with Ferumoxytol (250 μM) in the presence or absence of Ferrostatin-1 (Fer-1, 5 μM). **B.** Representative fluorescence images of intracellular labile Fe²⁺ detected by FerroOrange staining in PC-9 and H1915 cells after the indicated treatments, with quantitative analysis shown on the right. Hoechst was used for nuclear staining. Data are presented as mean ± SD from three independent experiments. ns, p ≥ 0.05; * p < 0.05; ** p < 0.01; *** p < 0.001.

**Fig. S2. Ferumoxytol enhances irradiation-induced lipid peroxidation and reduces clonogenic survival in PC-9 and H1915 cells. A.** Representative fluorescence images of lipid peroxidation detected by C11-BODIPY 581/591 staining in PC-9 and H1915 cells at 6 h after the indicated treatments, with quantitative analysis shown on the right. Oxidized signals are shown in green and reduced signals in red. Hoechst was used for nuclear staining. **B.** Representative clonogenic images of H1915 cells after the indicated treatments and exposure to 0, 1, 2, 4, or 6 Gy irradiation. Colonies were stained after 14 days, and surviving fractions were fitted using the single-hit multi-target model. The survival curves are shown on the right. Data are presented as mean ± SD from three independent experiments. * p < 0.05; ** p < 0.01; *** p < 0.001.

**Fig. S3. PC-9 validation of NCOA4 loss of function in the ferumoxytol-associated radiation response.** A. Representative western blot and densitometric analysis confirming siRNA-mediated NCOA4 knockdown in PC-9 cells. NCOA4 was normalized to GAPDH and expressed relative to the negative-control siRNA (NC) group. B. Representative immunoblots of NCOA4 and γ-H2AX in NC- or siNCOA4-transfected PC-9 cells after irradiation (IR) or IR plus ferumoxytol (IR+F). Cells were collected 2 h after 8 Gy irradiation. NCOA4 and γ-H2AX were normalized to GAPDH and expressed relative to NC+IR. C. Representative 4-Gy single-dose colony-formation images of NC- or siNCOA4-transfected PC-9 cells treated with IR or IR+F. One hundred cells were seeded per well, colonies containing at least 50 cells were counted after 14 days, and colony formation rate (%) was quantified on the right. Data are presented as mean ± SD from three independent experiments. ns, p ≥ 0.05; ** p < 0.01; *** p < 0.001; **** p < 0.0001.
